# What impact do assumptions about missing data have on conclusions? A practical sensitivity analysis for a cancer survival registry

**DOI:** 10.1186/s12874-017-0301-0

**Published:** 2017-02-06

**Authors:** M. Smuk, J. R. Carpenter, T. P. Morris

**Affiliations:** 1 Centre for Psychiatry, Queen Mary University of London, Charterhouse Sqaure, London, EC1M 6BQ UK; 2 Medical Statistics Department, London School of Hygiene and Tropical Medicine, London, UK; 3 London Hub for Trials Methodology Research, MRC Clinical Trials Unit at UCL, London, UK

**Keywords:** Missing data, Pattern-mixture model, Sensitivity analysis, Elicitation, Missing at random, Missing not at random

## Abstract

**Background:**

Within epidemiological and clinical research, missing data are a common issue and often over looked in publications. When the issue of missing observations is addressed it is usually assumed that the missing data are ‘missing at random’ (MAR). This assumption should be checked for plausibility, however it is untestable, thus inferences should be assessed for robustness to departures from missing at random.

**Methods:**

We highlight the method of pattern mixture sensitivity analysis after multiple imputation using colorectal cancer data as an example. We focus on the Dukes’ stage variable which has the highest proportion of missing observations. First, we find the probability of being in each Dukes’ stage given the MAR imputed dataset. We use these probabilities in a questionnaire to elicit prior beliefs from experts on what they believe the probability would be in the missing data. The questionnaire responses are then used in a Dirichlet draw to create a Bayesian ‘missing not at random’ (MNAR) prior to impute the missing observations. The model of interest is applied and inferences are compared to those from the MAR imputed data.

**Results:**

The inferences were largely insensitive to departure from MAR. Inferences under MNAR suggested a smaller association between Dukes’ stage and death, though the association remained positive and with similarly low *p* values.

**Conclusions:**

We conclude by discussing the positives and negatives of our method and highlight the importance of making people aware of the need to test the MAR assumption.

**Electronic supplementary material:**

The online version of this article (doi:10.1186/s12874-017-0301-0) contains supplementary material, which is available to authorized users.

## Background

The occurrence of missing values is inevitable in epidemiological and clinical research but often overlooked in publications [1]. Any analysis of incomplete data then makes assumptions about the missing data, intentional or unwitting. It is thus important to engage with the assumptions, consulting with experts in the substantive area, and feed these into analyses.

Multiple imputation (MI) is an increasingly popular tool for analysis of incomplete data, drawing several plausible values from an appropriate imputation distribution and combining results [2]. Software generally implements MI under the assumption of ‘Missing At Random’ (MAR) [3, 4]. This assumption states that the missing data mechanism is independent of the missing observations conditional on the observed data. If incorrect, analysis under MAR can be biased [5]. The MAR assumption is also inherently untestable and so it is critical to assess the sensitivity of inferences under alternative assumptions when data are assumed ‘Missing Not At Random’ (MNAR). The MNAR assumption states that the missing mechanism depends on the missing observations, even conditional on the observed data: the probability of missing observations depends on some unseen, underlying value. Inferences are generally more biased when the MNAR mechanism is dependent on the outcome verses covariate dependency [6], however this is not the only kind of MNAR mechanism [7].

In the statistical literature there are two broad approaches to analysing data under MNAR assumptions: a selection model [8, 9] or a pattern-mixture model [10]. A selection model contains a component which defines the probability of observations being missing and links this to the potentially missing variables. A pattern-mixture model creates a difference between the distributions of observed and missing data by specifying a distinct model for each pattern. These specified models can be then used to create MNAR inferences. When applying a pattern-mixture model in practice, one approach is to estimate the Bayesian predictive distribution for imputations under MAR but, before drawing the imputations, alter the distribution by a random draw from a prior distribution. The prior encodes specific assumptions about the difference between MAR and MNAR for this pattern. The result is imputed data with a mixture of potentially different imputations across different missing data patterns, hence the name ‘pattern-mixture’.

Carpenter and Kenward (2013) [11] found that the pattern-mixture approach is more readily understood by non-statistically trained experts than the selection approach as the assumptions can be represented graphically. However, standard statistical software and tutorials tend to assume MAR, and MI in practice tends to also assume MAR.

Routine use of Sensitivity Analysis (SA) in applied research is lacking and may be held back by lack of clear practical methods and examples. This paper aims to address these issues by providing such an example, lowering the barrier to more widespread adoption of SA.

Using colorectal cancer registry data, we explicitly perform analysis under departures from the MAR assumption. We describe pattern-mixture models, elicit beliefs from experts in the field, which are used to inform analyses under these MNAR beliefs, and implement MI under a pattern-mixture model where the Bayesian imputation model uses these priors.

## Methods

The National Cancer Data Repository (NCDR) [12] and the Hospital Episode Statistics (HES) [13] database provide our motivating dataset. The data were collected to “assess the variation in risk-adjusted 30-day postoperative mortality for patients with colorectal cancer between hospital trusts within the English NHS” (p.806) [14]. The dataset was comprised of individuals who underwent a major resection for their diagnosed primary colorectal cancer in any NHS English hospital between January 1998 and December 2006. These individuals were identified in the HES dataset and linked to the NCDR dataset to extract more detailed information.

The final dataset contains information on patient demographics, Dukes’ stage and 30-day postoperative mortality. Dukes’ cancer tumour stage is a measure of how far the cancer has spread, with four stages from A to D. Stage A is the least severe, meaning the cancer is only in the innermost lining of the bowel or slightly into the muscle, while stage D is the most severe, meaning the cancer has spread to other areas of the body.

The data consisted of 160,920 patients, of whom 10,704 (6.7%) died within 30 days after surgery. Data were complete for approximately 85% (136,040) of the patients. Missing observations occurred in three variables: Dukes’ stage had 15% of its observations missing; quintile index for multiple deprivation (IMD) had 0.25% missing; and the emergency admissions indicator (EAI) had 0.05% missing.

The aim of the study was to investigate risk-adjusted surgical outcome for patients with colorectal cancer at a population level described by Morris et al. (2011) [14]. We began by estimating the missing values under the assumption of MAR through MI by fully conditional specification (FCS) using Stata’s user-written program *ice* [4, 15]. The single level imputation model was chosen to match the model used in Morris et al. (2011) [14], to check that we could reproduce their results. We generated 10 imputed datasets (with 10 cycles). The imputation model included: postoperative mortality within 30 days (MORT), sex, hospital trust (organisation within NHS), median annual workload of each hospital trust, Dukes’ stage, IMD, age at diagnosis, year of diagnosis, year of operation, Charlson co-morbidity score, resection type (elective or emergency), EAI type (elective or emergency), cancer registry and site of initial primary tumour.

By default, the *ice* command assumes that missing observations are MAR. To apply sensitivity analysis we aim to alter the imputation assumptions to represent an MNAR mechanism.

To assess the sensitivity of the MAR assumptions, we compare the inferences from a multilevel binary logistic regression model created to analyse factors associated with 30-day postoperative mortality (substantive model) under different assumptions. This substantive model, also chosen to match that used in Morris et al.(2011) [14], had three hierarchical levels (level 1 patients, level 2 hospital trust and level 3 networks). The dependent variable is 30 day postoperative mortality and the covariates are age (per year increase), sex, site of the initial colorectal primary, IMD, year of diagnosis, Dukes’ stage at diagnosis, Charlson co-morbidity score and resection type.

We focus our sensitivity analysis on Dukes’ stage for simplicity, and because the missing information in IMD and EAI are negligible by comparison. Our approach is as follows:

1. Find predictors for Dukes’ stage being missing which are also strong predictors of Dukes’ stage.
2. Given each predictor from the previous step, we calculate the probability of being in each stage under the MAR assumption..
3. The above probabilities are then given to experts in a questionnaire. We elicit information from the experts by saying, ‘given these probabilities in the observed data what do you think are the probabilities in the missing data?’.
4. The estimated probabilities from the questionnaire are used to estimate the parameters of a Dirichlet distribution. Draws from the distribution are then used to impute under the MNAR assumption.
5. The substantive model is applied to the MNAR imputed data, inferences are compared to the MAR inferences to see how robust they are.

To identify possible predictors for Dukes’ stage being missing, we created a binary indicator for Dukes’ stage being missing and used it as an outcome in a logistic regression model, regressing it on all other available variables in a complete case analysis. We then used selected predictors to form a questionnaire to elicit information on the missing data distribution. Because this needs to be accessible, we do not consider UK National Health Service Trust, network, year of diagnosis, year of operation and medium annual workload for a trust as covariates in the model.

A backwards elimination procedure was used to select covariates out of the logistic regression, using a 1% level, with categorical variables with more than two categories tested using a joint parameter test. The final model had three covariates: age at diagnosis, 30-day postoperative mortality and tumour site. To reduce elicitation complexity we moved forward with the two most predictive covariates for missing Dukes’ stage, which were age at diagnosis (OR 0.92(0.91, 0.93) *p <* 0.001, per 10 years) and 30-day postoperative mortality (alive 30 days post-surgery: OR 1.84(1.76, 1.93) *p <* 0.001).

Age at diagnosis (AGE) was dichotomised as 0 when the patient is less than or equal to 70 years old and 1 otherwise. This was done as it would be extremely difficult to elicit information by year. We checked to see if AGE and MORT (1 if postoperative mortality within 30 days, other 0) were also good predictors of Dukes’ stage itself using a multinomial logistic regression. Both were strongly associated with the observed values of Dukes’ stage (*p <*0.001). Table 1 gives the proportion of missing Dukes’ stage data by AGE and MORT.

**Table 1:** Proportion (frequency) of missing observations in Dukes’ stage by dichotomised age and postoperative mortality

	30 Day Postoperative Mortality	
Age at Diagnosis	Alive	Deceased
Less than or equal to 70 years old	0.16 (11,453)	0.25 (548)
Greater than 70 years old	0.14 (10,539)	0.22 (1,894)

Table 1 shows that the majority of the missing observations occurred when patients are dead 30 days after surgery and ≤70 years old. As AGE and MORT are both binary variables, we label the cells in Table 1 by r for simplicity. Let r = 1 if AGE = 0 and MORT = 0, r = 2 if AGE = 0 and MORT = 1, r = 3 if AGE = 1 and MORT = 0 and r = 4 if AGE = 1 and MORT = 1.

To re-impute the data under an MNAR assumption we specified the probability of being in each Dukes’ stage given r. This was achieved by applying a multinomial logistic model with MORT and AGE as covariates for each imputed dataset and combining using Rubin’s rules.

$$ \begin{array}{cc}\hfill LOG\left(\frac{P\left( Duke{s}^{\hbox{'}} Stage\  j\right)}{P\left( Duke{s}^{\hbox{'}} Stage\ 1\right)}\right)={\alpha}_j+{\beta}_{j1} MORT+{\beta}_{j2} AGE\hfill & \hfill j=2,3,4\hfill \end{array}. $$

Hence:

$$ {\widehat{P}}_{i j}= P\left( Duke{s}^{\hbox{'}} Stag{e}_i= j\right)=\frac{{\left( exp\left({\alpha}_j+{\displaystyle {\sum}_{k=1}^2}{\beta}_{j k}{X}_{i k}\right)\right)}^{\left(1-{\delta}_{1 j}\right)}}{1+{\displaystyle {\sum}_{h=2}^4} exp\left({\alpha}_h+{\displaystyle {\sum}_{k=1}^2}{\beta}_{h k}{X}_{i k}\right)} $$

Where *i* = 1,..,160, 920 indexes patients, *j* = 1,…, 4 indexes the categories of Dukes’ stage, **X** is the covariate data and *δ*_1*j*_ = 1 if *j* = 1, or 0 otherwise. Here ***β*** and ***α*** are unknown parameters that can be estimated.

Next we can calculate the probability of being in a stage given *r* as ${\widehat{P}}_{rj}$:

$$ {\widehat{P}}_{rj}= E\left[{\widehat{P}}_{ij}\Big| r\right] $$

The probabilities ${\widehat{P}}_{rj}$ were used to elicit priors ${\widehat{\pi}}_{rj}$, representing experts’ opinions on the true distribution of Dukes’ stage for those with Dukes’ stage missing. To do this we created a questionnaire in Microsoft Excel. The questionnaires were sent out electronically and were accompanied by information on the data and sensitivity analysis concept (see Additional file 1 for details).

Having elicited prior beliefs, we computed the means *Ê*[*P*_*rjv*_] (denoted as ${\widehat{\pi}}_{rj}$) and variances $\widehat{Var}\left[{P}_{rjv}\right]$ (denoted as ${\widehat{V}}_{rj}$) of the opinions, where *r* = 1,…,4, *j* = 1,…,4 and *v* = 1,…,*K*, *K* is the number of experts. We used a Dirichlet prior distribution [16] because it is a conjugate prior of the categorical distribution (see Additional file 2 for details).

Values drawn from the Dirichlet with *γ*_*rj*_ = *E*[*S*_*rj*_] * *π*_*rj*_, were used to replace the MAR imputed values. Hence creating an MNAR assumption for the missing data as the observed and missing data distributions are no longer the same. The substantive model was applied to the MNAR imputed data using Rubin’s combining rules. The inferences were then compared to the inferences from the MAR imputed data and the subset of complete cases.

## Results

**Fig. 1:**
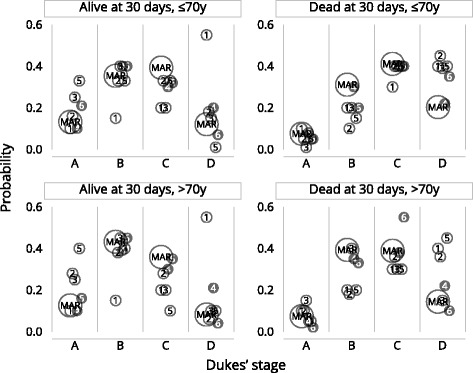
MAR probabilities and 6 reviewer estimates (reviewer 4 and 6 had not previously seen the data)

The questionnaire was completed by 6 people, 4 of whom had seen the study data as they had worked on the 2011 publication by Morris et al.[14]. The individual probability responses from the questionnaire and the ${\widehat{P}}_{rj}$ probabilities are graphically shown in Fig. 1, the two responders whom had not seen the study data have been represented by shaded circles.

**Fig. 2:**
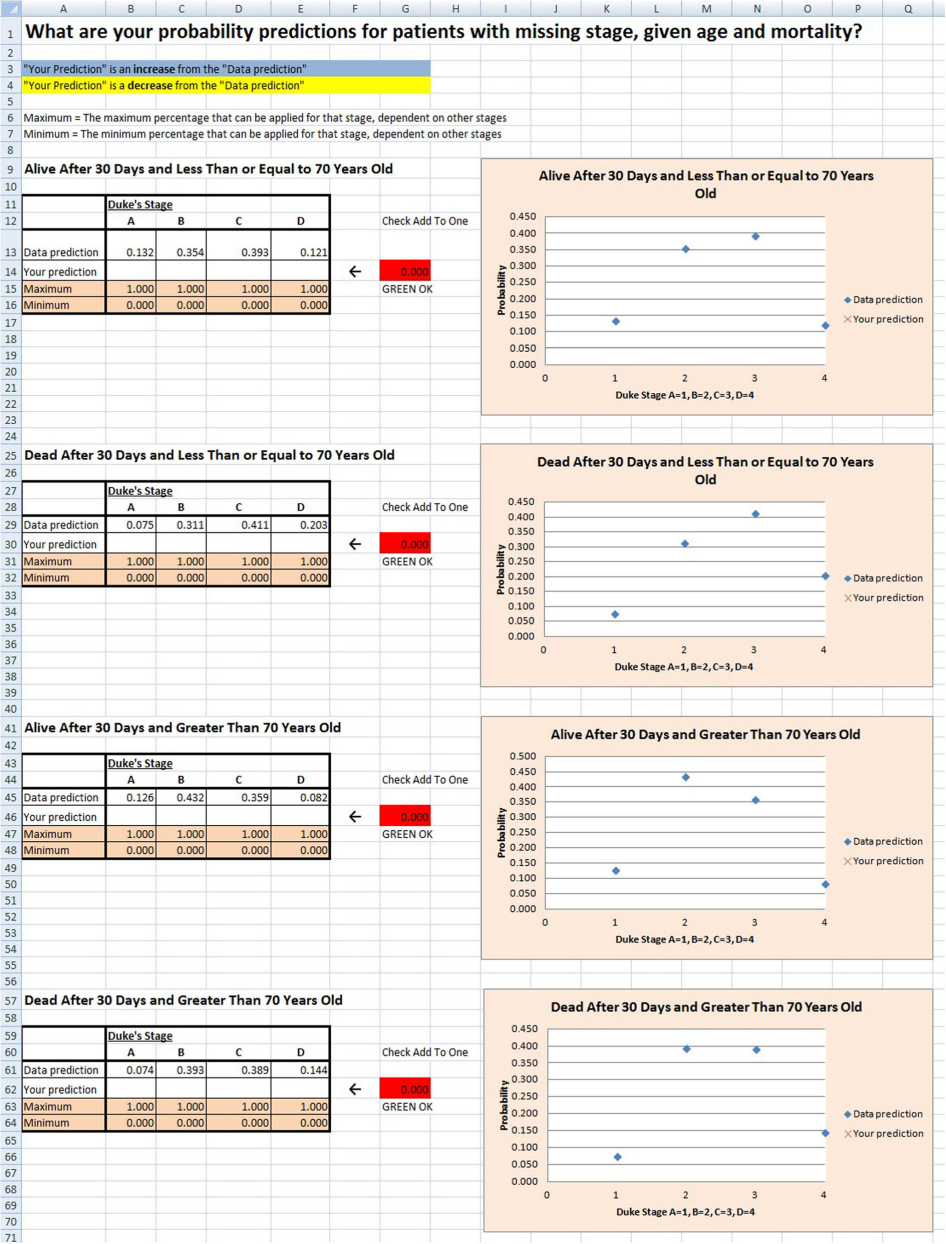
Screenshot of electronic questionnaire

**Fig. 3:**
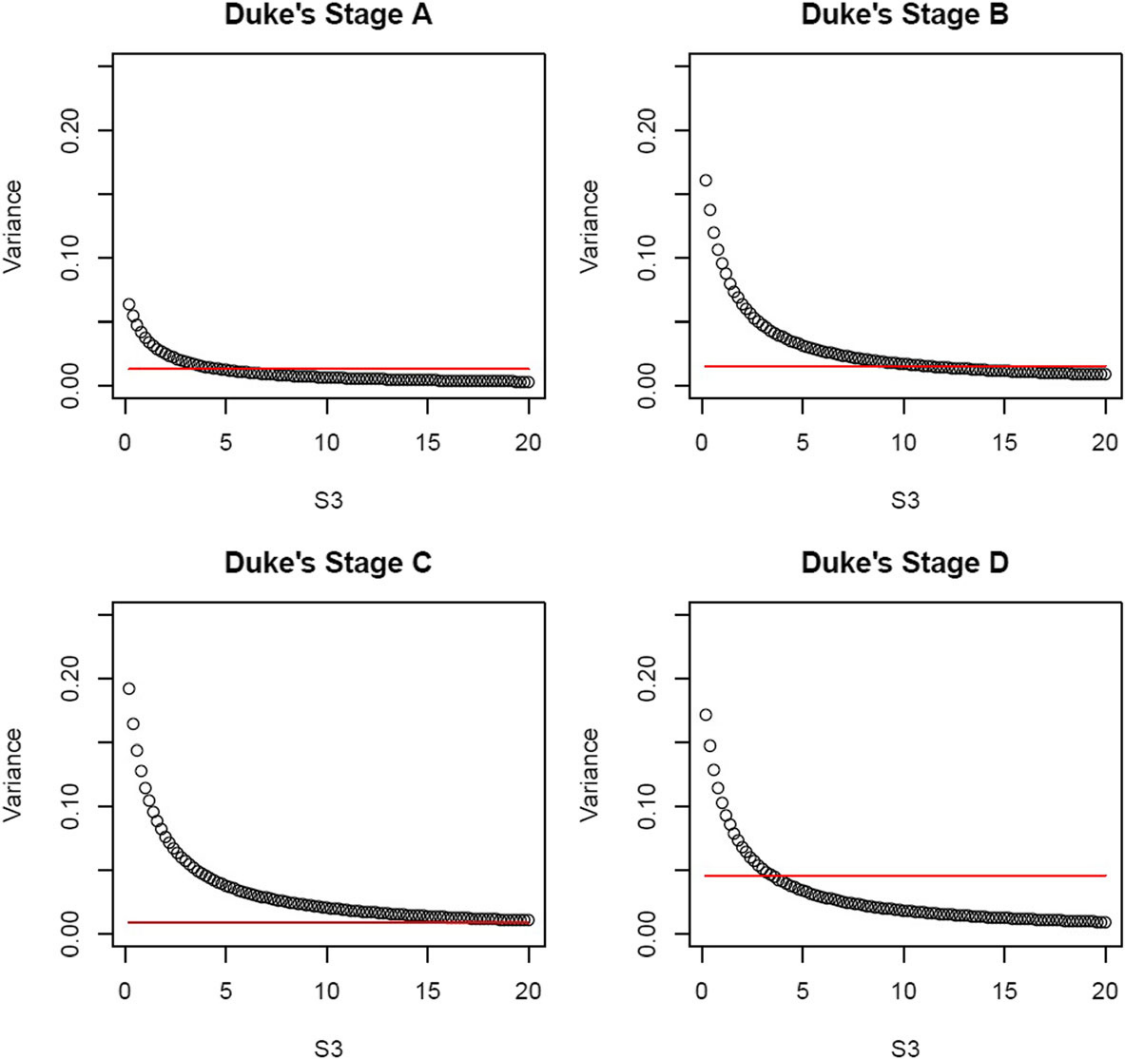
Dukes’ stage Dirichlet variances (circles) and empirical variance of responders (horizontal lines) for S_3_.

Figure 1 shows that the individual responses are quite varied. Questionnaire responders whom had seen the study data appear to be on average closer to the MAR estimate. The number of experts who completed the questionnaire was low as many experts replied saying they did not have the knowledge to give estimates for the probabilities being requested. The advantage that four of our sample had worked directly with the data meant they had a good understanding. However, this may mean that their probabilities were based on what they had seen in the data (data dependency) which may mean their elicited probabilities are unduly influenced by the published MAR analysis.

The means ${\widehat{\pi}}_{rj}$ and variances ${\widehat{V}}_{rj}$ from elicited probabilities for each Dukes’ stage and r are listed in Table 2.

**Table 2:** Dukes’ stage probabilities given age and mortality, estimated under MAR and the elicited mean (variance)

Characteristic	Dukes’ stage probabilities under MAR	Elicited Dukes’ stage probabilities Mean (variance)
Alive 30 days after surgery and age ≤ 70		
Dukes’ stage A	0.13	0.21 (0.008)
Dukes’ stage B	0.35	0.32 (0.010)
Dukes’ stage C	0.39	0.28 (0.005)
Dukes’ stage D	0.12	0.19 (0.045)
Dead 30 days after surgery and age ≤ 70		
Dukes’ stage A	0.08	0.05 (0.001)
Dukes’ stage B	0.31	0.17 (0.002)
Dukes’ stage C	0.41	0.38 (0.002)
Dukes’ stage D	0.20	0.40 (0.001)
Alive 30 days after surgery and age > 70		
Dukes’ stage A	0.13	0.24 (0.013)
Dukes’ stage B	0.43	0.37 (0.016)
Dukes’ stage C	0.36	0.23 (0.009)
Dukes’ stage D	0.08	0.17 (0.046)
Dead 30 days after surgery and age > 70		
Dukes’ stage A	0.07	0.08 (0.003)
Dukes’ stage B	0.39	0.26 (0.010)
Dukes’ stage C	0.39	0.36 (0.012)
Dukes’ stage D	0.14	0.29 (0.025)

Table 2 shows that the elicited probabilities for Dukes’ stage D given any r, are larger than those implied under MAR. The experts believe on average that the probability of being in Dukes’ stage D is higher than estimates derived from the observed data and imputation model. The elicited probabilities decrease Dukes’ stage B and C given any r and Dukes’ stage A tends to be increased for three of the four r categories.

The results from the multivariable analyses estimating the adjusted odds of death within 30 days of surgery are shown in Table 3.

**Table 3:** Adjusted odds ratios (AOR) for death within 30 days of surgery

	Multiple Imputation (MAR)			Multiple Imputation (MNAR)		
Characteristic	AOR	(95% CI)	*p* value	AOR	(95% CI)	*p* value
Age at diagnosis (per 10 years)	2.12	(2.07–2.17)	<0.001	2.08	(2.03–2.13)	<0.001
Year of diagnosis (per advancing year	0.97	(0.97–0.98)	<0.001	0.98	(0.97–0.98)	<0.001
Sex						
Female	1.00			1.00		
Male	1.21	(1.16–1.26)	<0.001	1.21	(1.16–1.26)	<0.001
Operation						
Elective	1.00			1.00		
Emergency	2.67	(2.53–2.82)	<0.001	2.76	(2.61–2.91)	<0.001
Dukes’ stage at diagnosis						
A	1.00			1.00		
B	1.24	(1.14–1.35)	<0.001	1.15	(1.04–1.26)	0.005
C	1.54	(1.42–1.68)	<0.001	1.29	(1.18–1.42)	<0.001
D	2.48	(2.25–2.73)	<0.001	1.82	(1.66–2.00)	<0.001
IMD income category						
1 (Most affluent)	1.00			1.00		
2	1.03	(0.96–1.10)	0.429	1.03	(0.96–1.10)	0.417
3	1.11	(1.04–1.19)	<0.001	1.12	(1.04–1.19)	0.002
4	1.21	(1.13–1.30)	<0.001	1.22	(1.14–1.30)	<0.001
5 (Most deprived)	1.32	(1.23–1.42)	<0.001	1.32	(1.23–1.42)	<0.001
Cancer site						
Colon	1.00			1.00		
Rectosigmoid	0.88	(0.82–0.96)	0.003	0.88	(0.82–0.96)	0.003
Rectum	0.94	(0.89–0.99)	0.018	0.91	(0.86–0.96)	0.001
Charlson comorbidity score						
0	1.00			1.00		
1	2.05	(1.94–2.17)	<0.001	2.06	(1.95–2.19)	<0.001
2	2.43	(2.25–2.62)	<0.001	2.42	(2.24–2.61)	<0.001
≥3	4.39	(3.99–4.83)	<0.001	4.35	(3.96–4.79)	<0.001

Table 3 shows that results from MAR and MNAR are broadly similar. The largest absolute differences in adjusted odds ratios (OR) can be observed for the Dukes’ stages. For Dukes’ stage B, the OR decreases by 7.3%, from 1.24 to 1.15; stage C decreases by 16.2%, from 1.54 to 1.29; and stage D decreases by 26.6%, from 2.48 to 1.82. It is important to note that the OR’s from the MAR imputation for Dukes’ stage C and D fall outside the confidence interval for the corresponding Dukes’ stage under MNAR, suggesting the OR’s differ however the direction of risk and *p* values remain the same. The imputation under the assumption of MNAR reduces the effect of Dukes’ stage on 30-day postoperative mortality. By contrast, the OR for elective vs. emergency surgery is 3.4% higher. This suggests that if the experts’ views are correct, Dukes’ stage is a less important predictor than suggested by the analysis assuming MAR, while emergency surgery is a more important predictor.

## Discussion

This paper has demonstrated one practical approach to sensitivity analysis which involves elicitation of opinions from experts and feeding these into a prior used to draw MI. The work was motivated by a colorectal cancer dataset collected and analysed by Morris et al. [14]. The publication handled the missing data by MI under MAR without performing any sensitivity analysis. Taking the substantive and imputation model from this their article, we replicated the results before extending the analysis, carrying out sensitivity analysis using pattern mixture models.

We received and used six experts’ responses to our questionnaire, which were modelled using a Dirichlet distribution to multiply impute data under the resulting distribution. We then applied the substantive model to each MNAR imputed data set and combined the results using Rubin’s rules. The elicited prior is useful as four of the six responders had a good knowledge of the data, however, more expert responses may have strengthened our conclusions.

Broadly speaking, the results were similar to those from the analysis under MAR. However, the effect of Dukes’ stage was reduced under MNAR, with estimates for Dukes’ stage C and D fully outside the MAR-based 95% confidence interval.

The method we have proposed and the associated software to implement it allows us to take into account informatively missing data in the analysis. This allows the sensitivity of inferences to the MAR assumption to be evaluated. The main difficulty of this method is in obtaining prior information that is reliable and accurate. First, we found not all experts were comfortable with giving their opinion, in line with [17]; second, it is unclear who exactly is an appropriate ‘expert’. We used electronic communication to elicit information from experts; however face to face meetings would almost certainly have aided this process as it would give more freedom to question and clarify concepts. Thus the accuracy of the information cannot be ascertained, more research into the reliability and methodology of collecting prior information is needed. Further research into the number of experts who need to be consulted should also be done, as it is not currently clarified in literature.

A further point of interest concerns the standard error of the parameter estimates under MNAR, and how they compare to those obtained under MAR. Ideally we would like them to be greater under MNAR, reflecting the extra uncertainty relative to MAR. However, this is not guaranteed under our approach - it depends on the variability of the imputation distribution derived from expert opinion relative to that under MAR. The standard errors in Table 3 suggest this is not an issue here.

Lastly, neither our MAR or MNAR imputation has allowed for the multilevel structure (multilevel by trust and registry) of the data and hence the clustering variability has not been incorporated, resulting in possible inference bias. This is because we wished to reproduce the inferences in Morris et al. [14], who did not use multilevel MI.

MI software is becoming more accessible and the default assumption for the missing data is MAR. If inferences are sensitive to this, those to whom the research is presented (e.g. journal readers) should be aware of this. As the MAR assumption is untestable, additional analysis to explore the sensitivity of inferences to the MAR assumption are desirable. However this extra work can be time consuming, so a limitation of our method is the time it takes to elicit the prior information possibly putting analysts off. However an advantage of this approach is that it is computationally simple once the prior has been elicited. Unlike under the selection modelling approach, the missingness mechanism model does not need to be jointly modelled with the substantive model, which can be computationally demanding.

In our setting we only selected two predictive variables, age at diagnosis and 30 day postoperative surgery mortality to frame the elicitation process. This was done to simplify the questionnaire. However in some situations this simplification would not be appropriate, making the elicitation of information difficult.

Of course, this approach is no substitute to collecting data fully. We do however believe that the process of eliciting information helps raise awareness of the potential loss of information and possible bias caused by missing data. This is turn leads to more emphases minimising missing data in future study designs.

## Conclusions

In summary, we believe the approach described here is a computationally feasible, accessible and practical approach to sensitivity analysis within epidemiology. We hope it may find application in the area where missing data are often an issue, because the data were collected for direct clinical need, not with research in mind.

Our research demonstrates a practical, computationally feasible, multiple-imputation based approach for investigating the robustness of scientific conclusions to the assumption that data are Missing at Random. We encourage readers faced with non-trivial proportions of missing data to consider this approach.

## Additional files

Additional file 1: Contains the questionnaire information used to elicit responses (Fig. 2). (DOCX 1055 kb)
Additional file 2: Contains information on the Dirichlet distribution and how to find the precision of the distribution so that the variance is similar to the questionnaire responses (Fig. 3). (DOCX 16 kb)

## Abbreviations

AGE: Dichotomised age at diagnosis
AOR: Adjusted odds ratio
EAI: Emergency admissions indicator
FCS: Fully conditional specification
HES: Hospital episode statistics
IMD: Quintile index for multiple deprivation
MAR: Missing at random
MI: Multiple imputation
MNAR: Missing not at random
MORT: Postoperative mortality within 30 days
NCDR: National cancer data repository
NHS: National health service
OR: Odds ratio
SA: Sensitivity analysis
