# Erratum to: What impact do assumptions about missing data have on conclusions? a practical sensitivity analysis for a cancer survival registry

**DOI:** 10.1186/s12874-017-0323-7

**Published:** 2017-03-29

**Authors:** M. Smuk, J. R. Carpenter, T. P. Morris

**Affiliations:** 1 Centre for Psychiatry, Queen Mary University of London, Charterhouse Square, London, EC1M 6BQ UK; 2 Medical Statistics Department, London School of Hygiene and Tropical Medicine, London, UK; 3 London Hub for Trials Methodology Research, MRC Clinical Trials Unit at UCL, London, UK

## Erratum

After the publication of the original article [1], it came to the author’s attention that an error affecting Figures 2 and 3, and the Additional files, has occurred.

During the author proofing stage, an instruction was made to move Figs. 2 and 3 to the Additional files. The instruction was misinterpreted by the Production team, resulting in the files being retained as Figs. 2 and 3, and the Additional files being subsequently incorrect.

The original article has now been updated to rectify this error.
