# Supplementary material for: What impact do assumptions about missing data have on conclusions? A practical sensitivity analysis for a cancer survival registry
Source: BMC Med Res Methodol. 2017 Feb 6;17:21. doi: 10.1186/s12874-017-0301-0 (PMC5294884; doi:10.1186/s12874-017-0301-0)

**Supplementary material:**

**Questionnaire Information for Sensitivity Analysis with Multiple Imputation**

**The Study**

The study aim is to investigate factors affecting thirty day postoperative mortality after colorectal cancer surgery in England. The data for this analysis consists of pooled data from eight population based cancer registries that cover England. This was matched with data from the hospital episode statistics data base extracting patients who “presented in any NHS hospital with a diagnostic code for cancer between April 1997 and June 2007. Information was extracted from this resource on all individuals who underwent a major resection for a primary colorectal cancer diagnosed between 1 January 1998 and 31 December 2006” [15]. There are 160,920 patients in the dataset of whom 85% have complete records. We aim to assess the sensitivity of the inferences to the assumptions about the reason for the missing data.

**Missing Data**

Three variables within the dataset contained missing observations. The variables are Dukes' stage (15% missing), index of multiple deprivation (0.25% missing) and emergency admission indicator (0.05% missing). The focus is the sensitivity of inferences to different assumptions about the missing Dukes' stage category (4 ordered categories, with A the least severe and D the most severe).

The default assumption is that the distribution of Dukes' stage, (i.e. the probability that a patient has a particular stage) given other covariates and postoperative mortality, is the same whether or not Dukes' stage is actually observed. This is known as the missing at random (MAR) assumption. The alternative is that, the probability that a patient has a particular stage, given other covariates and postoperative mortality differs between patients with Dukes' stage missing and observed.

**What we would like you to do**

We would like you to indicate your views about the probability a patient having a particular Dukes' stage among those where Dukes' stage is missing. We ask for probabilities separately for patients who did and did not survive 30 days after surgery, and for patients aged less than or equal to 70 and over 70.

In the attached spreadsheet, for each combination of age and mortality, we would like you to enter what you think the probability of Dukes' stage being A-D. Your probabilities must sum to 1 (check cell will turn green). To help you, the spreadsheet will automatically graph your probabilities alongside those predicted from the data.

As you fill in the probabilities, the peach colour cells tell you the maximum / minimum probabilities you can submit in for the particular cell. The cells will turn yellow if your entry is less than the data prediction or blue if it is greater than the data prediction.

**Helpful example**

For a patient with missing Dukes' stage who is under 70 and died within 30 days of surgery, the middle row of the Table below shows the probability, derived from the observed data, that they would have been diagnosed with Dukes' stage A, B, C or D. The third row of the table shows the corresponding probabilities, which an idiosyncratic clinician expected them to be.

|  | **Probability that Dukes’ stage is:** | | | | **Sum of A,B,C & D** |
| --- | --- | --- | --- | --- | --- |
|  | **A** | **B** | **C** | **D** |  |
| Derived from the data | 0.075 | 0.311 | 0.411 | 0.203 | 1 |
| Idiosyncratic clinician’s prediction | 0.010 | 0.090 | 0.550 | 0.350 | 1 |

Thus, while our prediction from the observed data is that 8% have Dukes' stage A, 31% stage B, 41% stage C and 20% stage D, the idiosyncratic clinician differs from this, giving 1% having stage A, 9% stage B, 55% stage C and 35% stage D.

If anything is not clear, or you have any comments then please contact Mel by email on

melanie.smuk@lshtm.ac.uk. Thank you for your help.

**Figure 3: Screen shot of electronic questionnaire.**


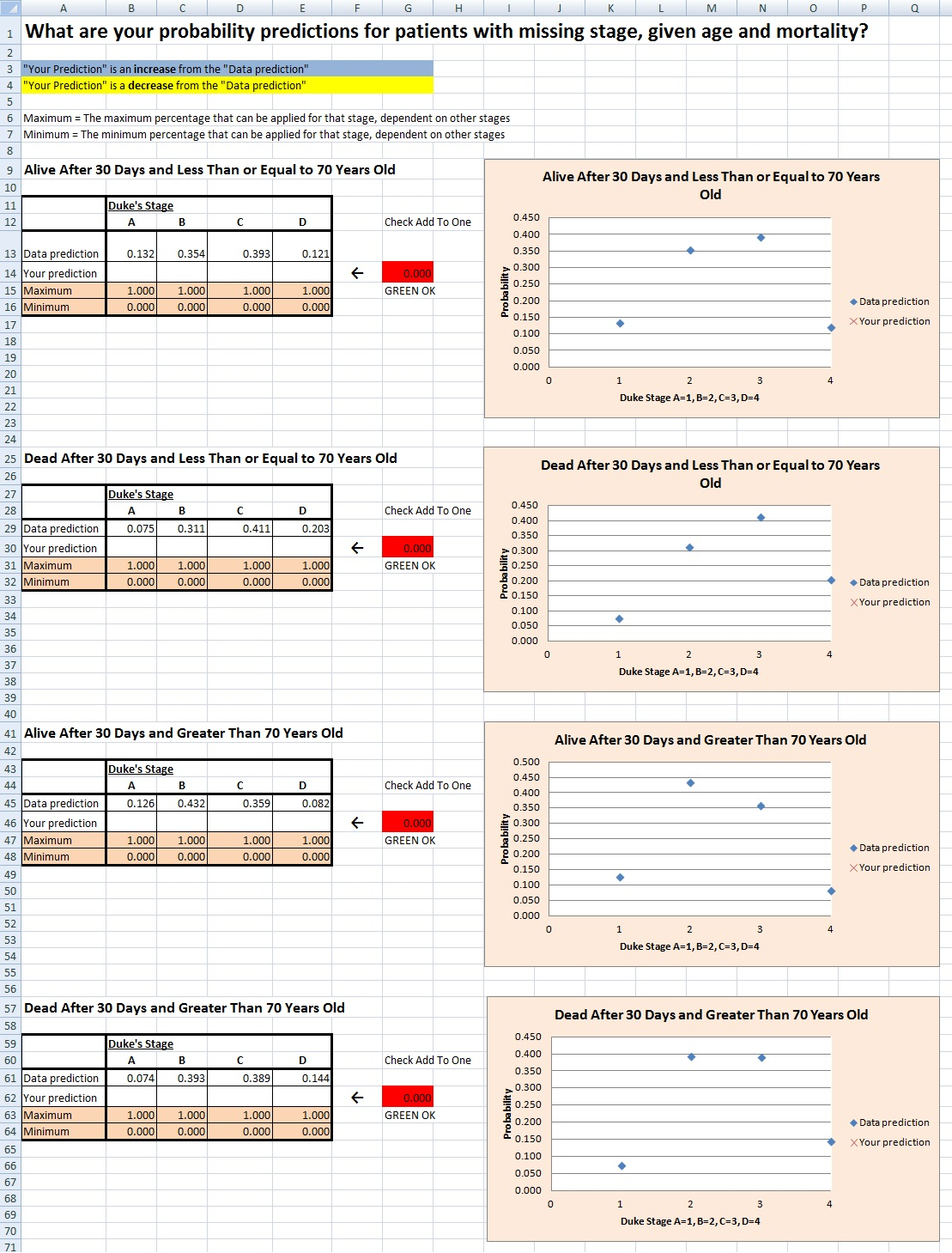

Supplement: Additional file 1: — Contains the questionnaire information used to elicit responses (Fig. 2). (DOCX 1055 kb) [file 12874_2017_301_MOESM1_ESM.docx]
