# Supplementary material for: What impact do assumptions about missing data have on conclusions? A practical sensitivity analysis for a cancer survival registry
Source: BMC Med Res Methodol. 2017 Feb 6;17:21. doi: 10.1186/s12874-017-0301-0 (PMC5294884; doi:10.1186/s12874-017-0301-0)
Supplement: Additional file 2: — Contains information on the Dirichlet distribution and how to find the precision of the distribution so that the variance is similar to the questionnaire responses (Fig. 3). (DOCX 16 kb) [file 12874_2017_301_MOESM2_ESM.docx]

**Appendix:**

**The Dirichlet distribution**

The Dirichlet distribution model is represented as:

$$f\left( \pi_{r1},\pi_{r2},\pi_{r3},\pi_{r4};\gamma_{r1},\gamma_{r2},\gamma_{r3},\gamma_{r4} \right)=\frac{1}{D\left( \gamma\right)}\prod_{j=1}^{4} \pi_{rj}^{\gamma_{rj}-1}.$$

Where $\pi_{rj}>0$, $\gamma_{rj}>0$ and $\sum_{j=1}^{4} \pi_{rj}=1$. The normalised constant $D\left( \gamma\right)$ is a multinomial beta function expressed in terms of a gamma function:

$$D\left( \gamma\right)=\frac{\prod_{j=1}^{4} \Gamma\left( \gamma_{rj} \right)}{\Gamma\left( \sum_{j=1}^{4} \gamma_{rj} \right)}.$$

The mean of the Dirichlet distribution is:

$$E\left[ \pi_{rj} \right]=\frac{\gamma_{rj}}{\sum_{s=1}^{4} \gamma_{rs}}=\frac{\gamma_{rj}}{S_{r}}.$$

(1)

The denominator $S_{r}$ defines the precision of the distribution. When scalar $S_{r}$ is large (much greater than 1), draws of $\pi_{rj}$ parameters are likely to be similar to their expectation $E\left[ \pi_{rj} \right]$, thus the distribution is more concentrated. When $S_{r}$ is small (less than 1), $\pi_{rj}$ parameter distribution is more diffuse.

The Dirichlet variance of $\pi_{rj}$ is:

$$Var\left[ \pi_{rj} \right]=\frac{\gamma_{rj}\left( S_{r}-\gamma_{rj} \right)}{S_{r}^{2}\left( S_{r}+1 \right)}.$$

(2)

We wished $Var\left[ \pi_{rj} \right]$ to be approximately equal to the empirical variance of the elicited data $\hat{V}_{rj}$, so when we draw from the Dirichlet distribution, the variance is similar to the responses in the questionnaire. We estimate $S_{r}$ to achieve this, using the method of moments. We used a range of values for $S_{r}$ in (1) (with $\hat{E}\left[ \pi_{rj} \right]=\hat{\pi}_{rj}$) which were then substituted into (2) to find the Dirichlet distribution variances $\hat{Var}\left[ \pi_{rj} \right]$. We then plotted these alongside the elicited data to help choose $S_{r}$. Figure 2 shows an example of finding S3 (the group of patients who are alive at 30 days and older than 70).

The solid lines in Figure 2 represent the empirical variances ($\hat{V}_{3j}$) of the estimates from the questionnaire responses. We wish the Dirichlet variance $\hat{Var}\left[ \pi_{3j} \right]$to be approximately equal to the questionnaire variance $\hat{V}_{3j}$. Clearly this is not achieved by any single S, however taking the average ($E\left[ \hat{S}_{3j} \right]$) of the $\hat{S}_{3}$'s when $\hat{Var}\left[ \pi_{3j} \right]$ equals the solid line $\hat{V}_{3j}$will solve (1) and (2).

Using this information we found the approximate parameters $\hat{\gamma}_{rj}$ for the Dirichlet distribution.

**(Figure 2 Here)**
